# Serum Endothelin-1 Correlates with Myocardial Injury and Independently Predicts Adverse Cardiac Events in Non-ST-Elevation Acute Myocardial Infarction

**DOI:** 10.1155/2020/9260812

**Published:** 2020-08-04

**Authors:** Anggoro Budi Hartopo, Indah Sukmasari, Ira Puspitawati, Budi Yuli Setianto

**Affiliations:** ^1^Department of Cardiology and Vascular Medicine, Faculty of Medicine, Public Health and Nursing Universitas Gadjah Mada-Dr. Sardjito Hospital, Yogyakarta, Indonesia; ^2^Department of Clinical Pathology and Laboratory Medicine, Faculty of Medicine, Public Health and Nursing Universitas Gadjah Mada-Dr. Sardjito Hospital, Yogyakarta, Indonesia

## Abstract

**Introduction:**

Serum endothelin-1 is increasingly released in acute myocardial infarction, by necrotic cardiomyocytes. In non-ST-elevation acute myocardial infarction (Non-STEMI), increased serum endothelin-1 on-admission may have clinical significance during acute hospitalisation events.

**Objective:**

The purpose of this study is to investigate whether increased serum endothelin-1 level predict adverse cardiac events in patients hospitalized with Non-STEMI.

**Methods:**

The design of this research was a prospective cohort study. Consecutive subjects with Non-STEMI undergoing symptom onset ≤24 hour were enrolled and observed during intensive hospitalization. Serum endothelin-1, troponin-I, and hs-C reactive protein were measured from peripheral blood taken on-admission. In-hospital adverse cardiac events were a composite of death, acute heart failure, cardiogenic shock, reinfarction, and resuscitated VT/VF.

**Results:**

We enrolled 66 subjects. The incidence of in-hospital adverse cardiac events is 13.6% (10 out of 66 subjects). Serum endothelin-1 level was significantly higher in subjects with in-hospital adverse cardiac events. Subjects with endothelin-1 level >2.59 pg/mL independently predicted adverse cardiac events in hospitalised Non-STEMI patients (adjusted odds ratio 44.43, 95% confidence interval: 1.44-1372.99, *p* value 0.03). The serum endothelin-1 level was correlated with serum troponin I level (correlation coefficient of 0.413, *p* value 0.012).

**Conclusion:**

Increased serum endothelin-1 on-admission correlated with increased troponin-I and independently predicted in-hospital adverse cardiac events in patients with Non-STEMI.

## 1. Introduction

Endothelin-1 is a functional peptide, which acts as a strong vasoconstrictor in the peripheral dan coronary vascular beds by stimulation of vascular smooth muscle cells [1, 2]. It also has a balancing vasodilator in both vascular beds via stimulation of nitric oxide [1, 2]. Therefore, it maintains the stability of vascular tones, in both systemic and coronary vasculatures [1, 2]. Endothelin-1 level in blood circulation is increased in acute myocardial infarction, an acute injurious event of myocardia due to reduced oxygenated blood supply by coronary arteries [3–5].

In patients with ST-elevation acute myocardial infarction (STEMI), increased circulating endothelin-1 associated with left ventricular systolic dysfunction and major complication during hospitalisation [6, 7]. Our previous study indicated that higher serum endothelin-1 measured on admission is predictive for in-hospital adverse cardiac events in patients with STEMI [8]. Increased circulating endothelin-1 associates with injury in coronary microcirculation during reperfusion strategy and increased incidence of major adverse cardiac events during hospitalisation, 30 days and 1 year after discharge in STEMI [9, 10].

In patients with Non-STEMI, in whom early coronary revascularisation could be withheld, the peak endothelin-1 level occurs within 24 hour after the onset of infarction and may persistent within 48 hours [3, 6]. The role of increasing circulating endothelin-1 in Non-STEMI during hospitalisation period has not yet been elucidated since a broad range of clinical severity in Non-STEMI and various revascularisation strategy based on in-hospital risk stratification. This study aims at investigating the association between the serum level of endothelin-1 and in-hospital adverse cardiac events in patients hospitalised with Non-STEMI.

## 2. Methods

The design of the research was a prospective cohort study, as previously published [8]. The subjects of the research were patients diagnosed as Non-STEMI and admitted in the intensive cardiac care unit (ICCU) of Dr. Sardjito General Hospital, Yogyakarta, Indonesia. The subjects were enrolled consecutively. We included subjects with a diagnosis of Non-STEMI; with the onset of anginal pain ≤24 hours prior to admission; and with age between 30 and 75 years old. We excluded subjects with a history of chronic heart failure with NYHA class ≥II, chronic kidney disease stage ≥III, hepatic cirrhosis, and malignancy; with concomitant infection and sepsis; with concomitant acute stroke and acute inflammatory state (such as acute arthritis and pericarditis) during hospitalisation; and with previous acute myocardial infarction (postinfarction angina). All subjects gave signed informed consent to participate in the study.

Upon admission, a peripheral venous blood sample was obtained from each subject during the supine position. The blood sample was centrifuged and introduced into a hematology analyzer for routine hematology examination and chemical analyzer for blood chemistry examination in the hospital central laboratory. Troponin I was measured by enzyme-linked fluorescent assay (ELFA) method with VIDAS (Biomerieux) (CV%: 5.667). The hs-CRP level was determined with the immunoturbidimetric method and analysed with Roche Cobas c501 (Roche Diagnostic, U.S.A) (CV%: 2.61). Another blood sample was centrifuged at 4000 r.p.m for 20 minutes and stored at −80°C freezer until analysis for endothelin-1 measurement. Endothelin-1 was detected and quantified with endothelin-1 immunoassay Quantikine^®^ ELISA kit (R&D Systems, Minneapolis, USA) according to manufacturer procedure instruction (CV%: 23). The ELISA method was performed once by a skilled technician.

The subject demographic and clinical data were collected during hospitalization. The treatments for subjects were in the discretion of attending cardiologists, without any interferences of this research. Subjects were observed from admission until hospital discharge or until adverse cardiac events occurred. The adverse cardiac event was the composite of cardiac death, acute heart failure, cardiogenic shock, reinfarction, and resuscitated ventricular arrhythmia. Cardiac death was fatal due to cardiac disease. Acute heart failure was defined as the occurrence of signs/symptoms of congestion and the use of intravenous diuretics. Cardiogenic shock was defined as the signs of reduced peripheral perfusion and the use of vasopressors drugs. Reinfarction was defined as the recurrent chest pain, recurrent ST-segment elevation, and an elevation of cardiac enzymes. Resuscitated ventricular arrhythmia was the return of spontaneous circulation after resuscitation for lethal arrhythmias. The subject enrollment, study protocol, biomarkers examination, and outcome measurement were in accordance to our previous study in subjects with STEMI [8].

The protocol of the research had been approved by the Medical and Health Research Ethics Committee Faculty of Medicine Universitas Gadjah Mada and Dr. Sardjito Hospital Yogyakarta, Indonesia.

For statistical analysis, the subjects were divided into two groups based on the presence of in-hospital adverse cardiac events. The normal distribution was tested with the Kolmogorov-Smirnov test. The comparison between normally distributed continuous data was performed with Student's *T*-test, while the Mann–Whitney test was used for not normally distributed continuous data. A comparison between categorical data was performed with the Chi-square test. A receiver operating characteristic (ROC) curve was designed to determine the cut-off point of endothelin-1 level to predict adverse cardiac events. A univariate and multivariable analysis with logistic regression test were performed to determine the independent predictor of an adverse cardiac event. A *p* value < 0.05 was set as statistical significance.

## 3. Result

**Figure 1:**
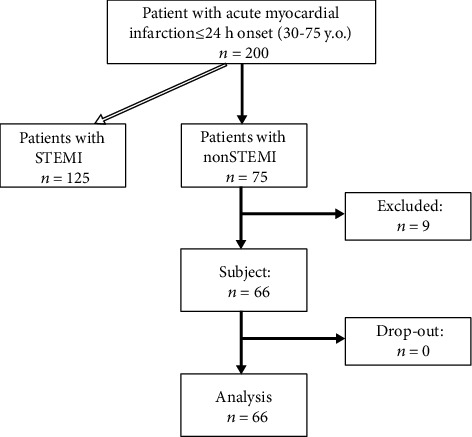
The flow-chart of subject enrollment, follow-up, and analysis. ^∗^Subjects with STEMI were analysed and published elsewhere [8].

Sixty-six subjects were enrolled in this study. The flowchart of the study subjects was shown in Figure 1. Mean endothelin-1 level in all subjects was 3.09 ± 1.9 pg/mL. In-hospital adverse cardiac events occurred in 10 subjects (15.2%). Subjects experienced in-hospital adverse cardiac events had significantly higher body mass index, a higher proportion of diabetes mellitus, lower systolic and diastolic pressures, higher heart rate, higher proportion of Killip class ≥ II, greater leucocytes counts, creatinine and glucose levels, and less beta-blocker usage (Table 1). Serum endothelin-1 level was significantly higher in subjects with in-hospital adverse cardiac events as compared with those without (4.16 ± 2.1 pg/mL vs. 2.90 ± 1.8 pg/mL, *p* = 0.04).

**Figure 2:**
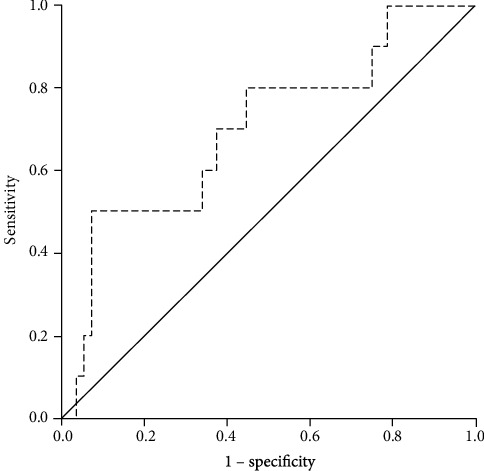
The ROC curve to determine the accuracy of endothelin-1 to predict adverse cardiac events (AUC 0.70), cut-off value 2.59 pg/mL (sensitivity 80%, specificity 55.4%).

The ROC curve indicated that serum endothelin-1 level has a predictive value for in-hospital adverse cardiac events with area under the curve of 0.70. Based on the ROC curve, the endothelin-1 level of 2.59 pg/mL had a sensitivity of 80% and specificity of 55.4% to accurately predict in-hospital adverse cardiac events (Figures 1 and 2). Subjects with endothelin-1 level > 2.59 pg/mL had higher proportion of in-hospital adverse cardiac events (80.0%) than those with endothelin-1 level ≤ 2.59 pg/mL (44.6%) (Table 1).

A multivariable analysis, by forward conditional logistic regression analysis, indicated that endothelin-1 level > 2.59 pg/mL independently predicted in-hospital adverse cardiac events (adjusted odds ratio 44.43, 95% confidence interval: 1.44-1372.99, *p* value 0.03). Other independent predictors for in-hospital adverse cardiac events were higher body mass index (adjusted odds ratio 1.32, 95% confidence interval: 1.01-1.73, *p* value 0.04), the presence diabetes mellitus (adjusted odds ratio 25.32, 95% confidence interval: 1.48-434.53, *p* value 0.03), and the less beta-blocker usage (adjusted odds ratio 0.13, 95% confidence interval: 0.01-0.38, *p* value 0.01). The univariate and multivariable analysis were shown in Table 2.

Among continuous variables, serum endothelin-1 level was correlated with serum creatinine level (correlation coefficient of 0.420, *p* value < 0.001) and troponin I level (correlation coefficient of 0.413, *p* value 0.012). Other variables, i.e., age, body mass index, anginal onset, systolic blood pressure, diastolic pressure, heart rate, hemoglobin level, leucocytes counts, platelet counts, glucose level, and hs-C reactive protein level, did not significantly correlate with serum endothelin-1 level (Table 3).

## 4. Discussion

The result of our study indicated that in patients with Non-STEMI underwent intensive care hospitalization, the increased on-admission serum endothelin-1 level independently associated with adverse cardiac events. The proportion of adverse cardiac events during intensive care was almost fivefold higher in patients with increased serum endothelin-1 level. Our study indicated cut-off value 2.59 pg/mL as the best value to independently predict in-hospital adverse cardiac events. The measurement of serum endothelin-1 level to predict in-hospital adverse cardiac event in Non-STEMI need to be further corroborated.

The incidence of Non-STEMI is much higher than in STEMI. Although in a short time period, the mortality rate is higher in STEMI, the long-term mortality is increased in Non-STEMI [11]. The pathomechanism of Non-STEMI is rather complex, not only the abrupt thrombus formation in the ruptured plaque but also involve several pathologies such as vulnerable plaque, coronary thrombosis, vulnerable characteristics of patients, endothelial dysfunction, accelerated atherothrombosis, secondary mechanisms, and myocardial injury [11]. Acute risk assessment is necessary by using Grace score, which includes age, systolic blood pressure, pulse rate, serum creatinine, Killip class at presentation, cardiac arrest at admission, elevated cardiac biomarkers, and ST deviation, to identify the risk of mortality while in hospital care, at 6 months discharge, 1 year, and lastly until 3 years follow-up [12]. Non-STEMI constitutes the non-ST-elevation acute coronary syndrome without acute thrombus occlusion, but with severe and complex coronary lesions and impaired myocardial perfusion due to microvascular dysfunction [13].

Expression of endothelin-1 is abundant in unstable and ruptured plaque. Vascular injury during acute myocardial infarction, and coronary interventional procedure, gives rise to increased endothelin-1 in coronary vasculature [14]. The impact of vasoconstriction and vascular dysfunction on endothelin-1 in coronary microvascular in Non-STEMI has been proven [15]. The use of endothelin-1 receptor antagonist reverse the endothelin-1 mediated coronary microvascular dysfunction [15]. While heparin and antiplatelets are advantageous to restore the microvascular blood flow, the excess of endothelin-1 may counteract this favourable property. In our study, despite all subjects had standard heparin and antiplatelets, those with higher endothelin-1 developed in-hospital adverse cardiac events.

Endothelin-1 may reflect systemic inflammation, in which occurred during Non-STEMI. In Non-STEMI without coronary revascularisation, the peak endothelin-1 level can reach 24 hours after the onset [3]. In patients undergoing coronary intervention, the endothelin-1 level may increase with a peak level at 1 hour [16]. In our study, the mean onset of anginal pain was 13 hours. This is the range of time before peak endothelin-1 level was achieved in Non-STEMI. In addition to vascular injury and dysfunction, the contribution of systemic inflammation and myocardial injury may be also significant. To support the notion, our study indicated the significant positive correlation between serum endothelin-1 with markers of myocardial injury, troponin I. However, the significant correlation was not observed between serum endothelin-1 and markers of systemic inflammation, leukocyte counts, and hs-C reactive protein level.

In conclusion, increased serum endothelin-1 on-admission correlated with increased troponin-I, a marker of myocardial injury, and independently predicted in-hospital adverse cardiac events in patients with Non-STEMI.

## 5. Limitation of Study

The limitations of this study are (1) a small sample size, which reduced the power of the study result, (2) the examination of biomarkers could be performed in all subjects, and (3) the follow-up period was in a too-short duration, which could be expanded into long-term period.

## Figures and Tables

**Table 1:** The characteristics of all subjects and groups based on the presence and absence of adverse cardiac events.

Characteristics	All subjects *n* = 66	Adverse cardiac events (+) *n* = 10	Adverse cardiac events (-) *n* = 56	*p* value
Demography				
Males, *n* (%)	53 (80.3)	9 (90.0)	44 (78.6)	0.40
Age (year)	57.8 ± 9.5	54.9 ± 9.2	58.3 ± 9.6	0.30
Body mass index, mean ± SD	25.0 ± 3.8	27.6 ± 4.2	24.6 ± 3.6	0.02
Diabetes mellitus, *n* (%)	20 (30.3)	7 (70.0)	13 (23.2)	<0.01
Hypertension, *n* (%)	42 (63.6)	6 (60.0)	36 (64.3)	0.53
Ischemic heart disease, *n* (%)	27 (40.9)	4 (40.0)	23 (41.1)	0.62
Dyslipidemia, *n* (%)	18 (27.3)	2 (20.0)	16 (28.6)	0.45
Clinics, mean ± SD				
Onset (hours)	13.5 ± 8.9	13.3 ± 9.9	13.6 ± 8.9	0.93
Systolic pressure (mmHg)	132.4 ± 22.5	115.2 ± 26.5	135.6 ± 20.5	<0.01
Diastolic pressure (mmHg)	79.4 ± 13.7	71.6 ± 16.8	80.8 ± 12.7	0.04
Heart rate (bpm)	77.2 ± 14.6	88.6 ± 17.9	75.1 ± 13.1	<0.01
Killip class ≥ II, *n* (%)	8 (12.1)	5 (50.0)	3 (5.4)	<0.01
Laboratory, mean ± SD				
Hemoglobin (g/dL)	13.5 ± 2.0	14.5 ± 1.8	13.3 ± 1.9	0.10
Leucocytes (×10^3/^mm^3^)	9.5 ± 2.9	12.1 ± 3.5	8.9 ± 2.5	<0.01
Platelets (×10^3/^mm^3^)	235.8 ± 51.3	217.0 ± 47.6	239.2 ± 51.4	0.21
Creatinine (mg/dL)	1.2 ± 0.5	1.8 ± 1.0	1.1 ± 0.4	<0.01
Glucose (mg/dL)	168.7 ± 91.9	233.3 ± 113.9	156.8 ± 83.1	0.01
Troponin I (ng/mL)	0.5 (0.1-2.9)	2.4 (0.1-4.9)	0.47 (0.06-2.4)	0.29^∗^
hs-C reactive protein (mg/L)	3.1 (0.9-8.5)	2.6 (0.8-5.8)	11.1 (2.1-61.4)	0.07^∗^
Treatment strategy, n (%)				
Coronary intervention	26 (39.4)	5 (50.0)	21 (37.5)	0.34
Unfractionated heparin	50 (75.8)	10 (100.0)	40 (80.0)	0.15
LMWH/Fondaparinux	16 (24.2)	0 (0.0)	16 (20.0)	0.15
ACE-I/ARB	62 (93.9)	10 (100.0)	52 (92.9)	0.51
Beta blocker	45 (68.2)	3 (30.0)	42 (75.0)	<0.01
Coronary artery disease (*n* = 31)				
1 vessel disease	2 (6.3)	0 (0.0)	2 (7.7)	0.78
2 vessel disease	10 (31.3)	1 (20.0)	9 (34.6)	
3 vessel disease	19 (59.4)	4 (80.0)	15 (57.7)	
Endothelin-1 level (pg/mL)	3.09 ± 1.9	4.16 ± 2.1	2.90 ± 1.8	0.04
Endothelin-1 level > 2.59 pg/mL	33 (50.0)	8 (80.0)	25 (44.6)	0.04

^∗^Mann-Whitney test (troponin I (*n* = 36); hs-C reactive protein (*n* = 31)).

**Table 2:** Univariate and multivariable analysis of covariables, which predict in-hospital adverse cardiac events.

Covariables	Unadjusted OR (95% CI)	*p* value	Adjusted OR (95% CI)	*p* value
Body mass index	1.19 (1.01-1.42)	0.04	1.32 (1.01-1.73)	0.04
Diabetes mellitus	7.72 (1.74-34.18)	<0.01	25.32 (1.48-434.53)	0.03
Systolic pressure	0.95 (0.92-0.99)	0.01	N/A	0.58
Diastolic pressure	0.95 (0.91-1.01)	0.05	N/A	0.66
Heart rate (bpm)	1.07 (1.01-1.12)	0.01	N/A	0.32
Killip class ≥ II, *n* (%)	17.67 (3.23-96.69)	<0.01	N/A	0.58
Leucocytes	1.42 (1.10-1.83)	<0.01	N/A	0.08
Creatinine (mg/dL)	10.34 (1.68-63.82)	0.01	N/A	0.66
Glucose (mg/dL)	1.01 (1.00-1.01)	0.03	N/A	0.47
Beta blocker	0.14 (0.03-0.63)	0.01	0.13 (0.01-0.38)	0.01
Endothelin-1 > 2.59 pg/mL	4.96 (1.01-25.48)	0.04	44.43 (1.44-1372.99)	0.03

N/A: not applicable.

**Table 3:** Correlation between endothelin-1 level with other continuous variables.

Variables	Coefficient correlation	*p* value
Age	0.109	0.38
Body mass index	-0.055	0.68
Onset	0.023	0.86
Systolic pressure	-0.218	0.08
Diastolic pressure	-0.150	0.233
Heart rate	0.213	0.089
Hemoglobin	-0.068	0.590
Leucocytes	0.214	0.084
Platelets	-0.060	0.631
Creatinine	0.420	<0.001
Glucose	0.087	0.494
Troponin I	0.413	0.012^∗^
hs-C reactive protein	0.306	0.094^∗^

^∗^Spearman correlation test (troponin I (*n* = 36); hs-C reactive protein (*n* = 31)).

## Data Availability

The data used to support the findings of this study are available from the corresponding author upon request.

## References

[1] Yanagisawa M., Kurihara H., Kimura S. (1988). A novel potent vasoconstrictor peptide produced by vascular endothelial cells. *Nature*.

[2] Kinlay S., Behrendt D., Wainstein M. (2001). Role of endothelin-1 in the active constriction of human atherosclerotic coronary arteries. *Circulation*.

[3] Wieczorek I., Haynes W. G., Webb D. J., Ludlam C. A., Fox K. A. (1994). Raised plasma endothelin in unstable angina and non-Q wave myocardial infarction: relation to cardiovascular outcome. *British Heart Journal*.

[4] Qiu S., Th&eacute;roux P., Marcil M., Solymoss C. (1993). Plasma endothelin-1 levels in stable and unstable angina. *Cardiology*.

[5] Vojáček J., Kolář J., Lisý O. (1999). Time course of endothelin-1 plasma level in patients with acute coronary syndromes. *Cardiology*.

[6] Stewart D. J., Kubac G., Costello K. B., Cernacek P. (1991). Increased plasma endothelin-1 in the early hours of acute myocardial infarction. *Journal of the American College of Cardiology*.

[7] Katayama T., Yano K., Nakashima H. (2005). Clinical significance of acute-phase endothelin-1 in acute myocardial infarction patients treated with direct coronary angioplasty. *Circulation Journal*.

[8] Setianto B. Y., Hartopo A. B., Sukmasari I., Puspitawati I. (2016). On-admission high endothelin-1 level independently predicts in-hospital adverse cardiac events following ST-elevation acute myocardial infarction. *International Journal of Cardiology*.

[9] Freixa X., Heras M., Ortiz J. T. (2011). Usefulness of endothelin-1 assessment in acute myocardial infarction. *Revista Española de Cardiología*.

[10] Omland T., Lie R. T., Aakvaag A., Aarsland T., Dickstein K. (1994). Plasma endothelin determination as a prognostic indicator of 1-year mortality after acute myocardial infarction. *Circulation*.

[11] Luscher T. F., Barton M. (2000). Endothelins and endothelin receptor antagonists. *Circulation*.

[12] Thygesen K., Alpert J. S., Jaffe A. S. (2012). Third universal definition of myocardial infarction. *Circulation*.

[13] Petronio A. S., Amoroso G., Limbruno U. (1999). Endothelin-1 release from atherosclerotic plaque after percutaneous transluminal coronary angioplasty in stable angina pectoris and single-vessel coronary artery disease. *The American Journal of Cardiology*.

[14] Wong G. C., Morrow D. A., Murphy S. (2002). Elevations in troponin T and I are associated with abnormal tissue level perfusion: a TACTICS-TIMI 18 substudy. Treat angina with Aggrastat and determine cost of therapy with an invasive or conservative strategy-thrombolysis in myocardial infarction. *Circulation*.

[15] Guddeti R. R., Prasad A., Matsuzawa Y. (2016). Role of endothelin in microvascular dysfunction following percutaneous coronary intervention for non-ST elevation acute coronary syndromes: a single-centre randomised controlled trial. *Open Heart*.

[16] Cuculi F., Lim C. C. S., van Gaal W. (2011). Systemic levels of endothelin correlate with systemic inflammation and not with myocardial injury or left ventricular ejection fraction in patients undergoing percutaneous coronary intervention and on-pump coronary artery bypass grafting. *Interactive Cardiovascular and Thoracic Surgery*.

